# Temporal-insular spreading time in temporal lobe epilepsy as a predictor of seizure outcome after temporal lobectomy

**DOI:** 10.1097/MD.0000000000030114

**Published:** 2022-08-19

**Authors:** Xi Zhang, Guojun Zhang, Tao Yu, Cuiping Xu, Jin Zhu, Xiaoming Yan, Kai Ma, Runshi Gao

**Affiliations:** a Beijing Institute of Functional Neurosurgery, Department of Functional Neurosurgery, Xuanwu Hospital, Capital Medical University, Beijing, China; b Beijing Children’s Hospital, Capital Medical University, Beijing, China.

**Keywords:** anterior temporal lobectomy, epilepsy surgery, insula, SEEG, temporal lobe epilepsy

## Abstract

Insular involvement in temporal lobe epilepsy (TLE) has gradually been recognized since the widespread use of stereoelectroencephalography (SEEG). However, the correlation between insular involvement and failed temporal lobe surgery remains unclear. In this study, we analyzed the surgical outcomes of TLE patients who underwent temporal and insular SEEG recordings and explored the predictors of failed anterior temporal lobectomy (ATL) in these patients with temporal seizures.

Forty-one patients who underwent ATL for drug-resistant TLE were examined using temporal and insular SEEG recordings. The clinical characteristics, SEEG data, and postoperative seizure outcomes of these patients were analyzed, and multivariate analysis was used to identify the predictors of surgical outcome.

In this series, the ictal temporal discharges invaded the insula in 39 (95.1%) patients. Twenty-three (56.1%) patients were seizure-free (Engel class I) after ATL with at least 1 year follow-up. Only temporal-insular spreading time (TIST) was an independent predictor of postoperative seizure-free outcomes (*P* = .035). By creating receiver operating characteristic curves for TIST, 400 milliseconds was identified as the cutoff for classification. All patients were classified into 2 groups (TIST ≤ 400 milliseconds and TIST > 400 milliseconds) based on the cutoff value; the difference in seizure-free rates between the 2 groups was significant (*P* = .001).

The very early insular involvement in TLE may be associated with poorer seizure outcomes after ATL. Our findings may be helpful for estimating the appropriate operative procedures and will be valuable for evaluating the prognosis of TLE patients with temporal-insular SEEG recordings and temporal lobectomy.

## 1. Introduction

Surgery has proven to be effective for most patients with drug-resistant temporal lobe epilepsy (TLE).^[1,2]^ However, about 20% to 30% of the intractable TLE patients continue to have seizures after a temporal lobectomy.^[3,4]^ Some studies have revealed that insular involvement in mesiotemporal lobe epilepsies could explain some persistent seizures after anterior temporal lobectomy (ATL).^[5,6]^ In these patients, insular involvement is considered a potential risk factor for surgical failures.^[6–8]^

The insula and temporal lobe (TL) belong to the limbic system and have very close fiber connections. The symptoms evoked by insular cortex stimulation are similar to those observed during TL seizures.^[9]^ Stereoelectroencephalography (SEEG) studies showed that the insula is frequently invaded by TL seizures, suggesting that insular seizures might explain some failures after temporal lobectomy.^[10]^ Previous studies have shown that the surgical outcome of TLE patients without insular lobe involvement may be better than that of TLE patients with insular spread.^[11]^ However, the extent to which the insular involvement influences surgical treatment in TLE remains an unanswered question.

We performed a retrospective study of 41 patients identified as having TLE based on the SEEG recordings. All patients underwent unilateral or bilateral temporal-insular SEEG implantation, and intracranial electrodes were used to exclude independent insular seizures and to evaluate insular cortex involvement during temporal seizures. Based on SEEG recordings, we tried to analyze the correlation between insular involvement and the surgical outcome of TLE, and whether the clinical onset features, neuroimaging data, and SEEG propagation mode were helpful for predicting seizure freedom outcomes.

## 2. Methods

### 2.1. Patients

This series consisted of 41 patients who underwent SEEG recordings at Xuanwu Hospital for the presurgical evaluation of epilepsy between January 2017 and December 2019 and had a minimum of 1-year postoperative follow-up. The patient selection criteria included

1. focal onset with impaired awareness,
2. focal lesions in the TL, or negative magnetic resonance imaging (MRI) findings,
3. intracerebral electrodes were implanted in the temporal and insular cortices, and
4. underwent ATL.

The all-surgical specimens were submitted for histopathological examination. This study was approved by the Ethics Committee of Xuanwu Hospital, Capital Medical University, Beijing, China. Written informed consent was obtained from all patients for being included in the study, or a parent/legal guardian of patients under the age of 18 years.

### 2.2. Presurgical evaluation and seizure data

All patients underwent a comprehensive evaluation, including detailed seizure history, neurological examination, neuropsychological testing, scalp video-EEG, MRI, magnetoencephalography, and 18-fluorodeoxyglucose positron emission tomography.

Seizure-related data were collected during the presurgical examination; a history of tonic-clonic seizures including bilateral tonic-clonic seizures was collected based on both the medical history and preoperative video-EEG recordings. The seizures result was obtained from a postoperative follow-up database and categorized using the International League Against Epilepsy surgical categorization method.

### 2.3. SEEG investigation

SEEG electrode implantations were performed after noninvasive assessment. Recordings were performed with intracranial electrodes (Sinovation Medical Technology, Beijing, China; 8-20 stainless steel contacts, diameter: 0.8 mm, contact length: 2 mm, 1.5 mm apart) implanted in stereotactic conditions.

The implantation of the electrodes was guided by a robotic arm (Sinovation, SR1, Beijing, China) driven by stereotactic software. The target area of the electrodes was determined by noninvasive evaluation and hypotheses regarding the epileptogenic area and discharge spreading. The mesial and lateral TLs, insular cortex, and suprasylvian and infrasylvian opercular cortices were the focus areas based on the preoperative evaluation results. A total of 308 electrodes were implanted in 41 patients (4–12/patient, mean: 7.6), bilateral implantation in 12 patients, and unilateral implantation in 29 patients. The insula was recorded using 1 to 5 electrodes in each patient.

The SEEG data were analyzed using visual electrophysiologic analysis in the study. The ictal onset of SEEG was visually defined, and the ictal patterns often consist of high-frequency low-amplitude spike discharges or of the fast synchronizing discharges.^[10]^ The insular involvement was identified as the first clear background SEEG change that occurred posterior to the onset of the temporal seizure. The time interval between the onset of TL and insular involvement can be measured, and at least 3 conventional seizures were analyzed.

### 2.4. Statistical methods

Continuous variables were described using means ± standard deviations. Categorical variables were described using absolute numbers and percentages. Chi-square and logistic regression were used, conducted with SPSS for Windows Version 22 (SPSS Inc, Chicago, IL). A significant *P* value was established at <.05.

Chi-square and Fisher exact tests were used for univariate analyses to examine relationships between categorical variables. Binary logistic regression was performed to identify variables that could predict the outcome independently. To describe the predictive ability of the variables proven to be independent predictors in this study, we constructed receiver operating characteristic (ROC) curves, and the cutoff values were determined according to the ROC curves.

## 3. Results

### 3.1. Patient population

The 41 patients included 25 male and 16 female patients, aged 14 to 42 years with a median age of 26 years; the mean age at epilepsy onset was 16.3 ± 8.2 years, the mean duration of epilepsy was 10.2 ± 7.4 years, and the mean age at surgery was 28.2 ± 10.5 years. There were 12 of the 41 patients (29.3%) who demonstrated MRI signs of hippocampal sclerosis (HS). All patients in this study underwent SEEG recording, and 103 spontaneous seizures were analyzed. In this group, seizures originated from the TL in all the 41 patients, 39 (95.1%) of whom had discharges invading the insula. The clinical characteristics of all the patients are summarized in Tables 1 and 2.

**Table 1:** Patients’ characteristics and univariate analysis of risk factors of seizure recurrence.

Variables	N (%)	Seizure-free/nonseizure-free (n)	*χ* ^2^	*P* value
All patients	41	19/22		
Gender			1.071	.452
Male	25 (60.9)	12/13		
Female	16 (39.1)	7/9		
History of febrile seizures during childhood			3.223	.311
Yes	9 (22.0)	5/4		
No	32 (78.0)	14/18		
MRI signs of hippocampal sclerosis			4.803	.057
Yes	12 (29.3)	9/3		
No	29 (70.7)	10/19		
Focal to bilateral tonic-clonic seizures			8.786	.044
Yes	13 (31.7)	4/9		
No	28 (68.3)	15/13		
Vegetative symptoms			3.037	.395
Yes	12 (27.9)	5/7		
No	29 (72.1)	14/15		
Respiratory symptoms			1.331	.638
Yes	6 (14.6)	2/4		
No	35 (85.4)	17/18		
Somatosensory symptoms			1.447	.601
Yes	8 (19.5)	5/3		
No	33 (80.5)	14/19		
Simple motor signs			9.204	.006
Yes	5 (12.2)	1/4		
No	36 (87.8)	18/18		

MRI = magnetic resonance imaging.

**Table 2:** Binary logistic regression of the continuous variables.

Variables	Mean ± SD (min–max)	*β*	*P* value	OR
Age at surgery (yr)	28.2 ± 10.5 (11−43)	0.012	.450	0.987
Epilepsy duration (yr)	10.2 ± 7.4 (4−31)	−0.153	.744	0.829
Temporal-insular spreading time (ms)	4950.1 ± 9630.7 (100–30,000)	−0.103	.017	0.983

OR = odds ratio, SD = standard deviation.

### 3.2. SEEG recordings

SEEG-recorded interictal activity usually consists of a rhythm oscillation at a frequency of 6 to 12 Hz, and 16 patients showed interictal spikes or rhythmic spikes in the insular cortex that were not synchronous with hippocampal activity. In most cases, the spikes or spike waves were recorded in a focal gyrus of the insula.

**Figure 1.**
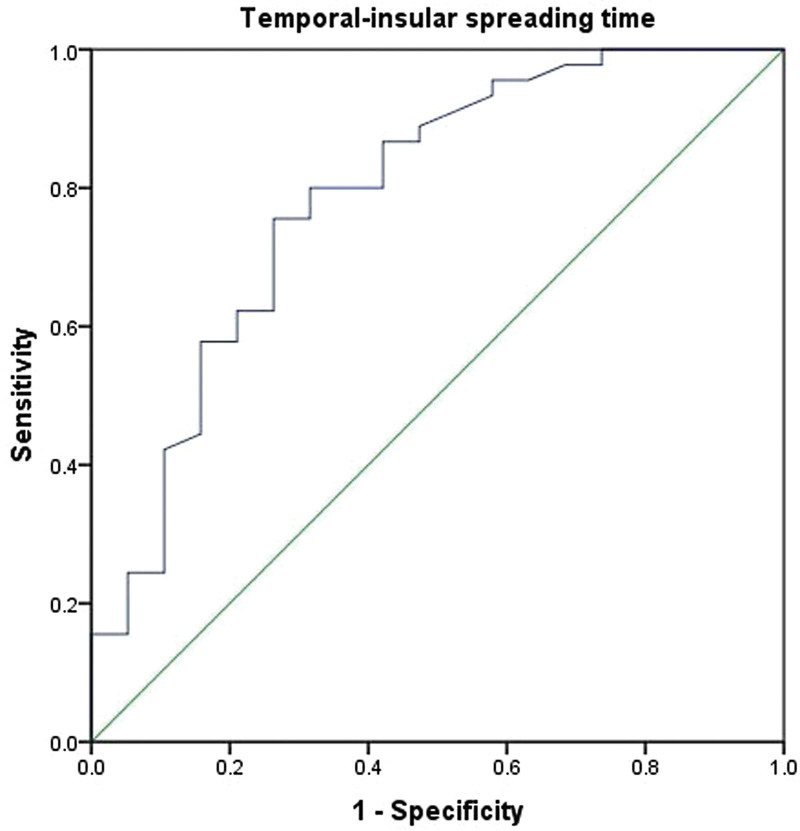
Stereoelectroencephalography recordings of seizure onset in patient 12. A very early propagation of fast discharge (A) at ictal onset and a subsequent low-voltage fast discharge (B) are observed on the insular contacts.

The ictal temporal discharges invading the insula were noticed in 39 (95.1%) patients, with a mean temporal-insular spreading time (TIST) of 4950.1 ± 9630.7 milliseconds (range 100 ms–30 s). The TIST ranged from 0 to 400 milliseconds in 9 patients, 400 to 30,000 milliseconds in 30 patients, and no obvious temporal-insular involvement in 2 patients. Three main spreading patterns were observed:

1. spikes and multi-spike waves invading the insula after a TIST (150 milliseconds–30 seconds) of temporal onset in 26 cases;
2. the low-voltage fast discharge activities spread quickly from the TL to the insula with a TIST shorter than 500 milliseconds in 9 cases (Fig. 1);
3. a rhythmic slow-wave discharge was recorded in the insula that occurred after the clinical onset in 6 cases.

The ictal temporal discharges propagated to the insular cortex mainly followed 3 spreading routes: the external temporal neocortex discharge invaded the insular cortex after passing through the mesial structures in 22 (53.7%) cases, the ictal discharges spread from the external temporal cortex to the insula in 8 (19.5%) cases, and ictal discharges directly invaded the insula from the mesial structures in 4 (9.8%) cases.

Twelve of 41 patients underwent bilateral temporal-insular SEEG coverage, and ictal discharges propagated to the contralateral hemisphere in 10 of 12 patients. The temporal discharges directly invaded the contralateral hippocampus and insular cortex in 6 cases; the discharges invaded the ipsilateral insula and subsequently propagated to the contralateral insula or TL in 4 cases. Ictal discharges originated in the temporal pole and then spread to the ipsilateral orbitofrontal cortex in the remaining 2 cases.

### 3.3. The ictal semiology

Symptoms associated with insular involvement were observed in 26 of the 41 patients. The respiratory symptoms, such as suffocation in the throat or dyspnea, were observed in 6 cases (14.6%); somatosensory symptoms in the trunk or limbs, such as numbness or acmesthesia, were observed in 8 cases (19.5%); vegetative symptoms (salivation, tachycardia, piloerection) were observed in 12 cases (29.3%). Besides, there were also some simple motor signs that were observed at the early stage of seizures, such as face or arm clonic (tonic spasm of face and upper limb), were observed in 5 cases (12.2%).

### 3.4. Factors influencing surgical outcome

In univariate analyses, 3 variables showed a significant association with nonseizure-free outcomes: focal to bilateral tonic-clonic seizures (*P* = .044), simple motor signs (*P* = .006), and TIST (*P* = .017). None of the other factors was associated with postoperative seizure outcomes (Tables 1 and 2).

In multivariate analyses, binary logistic regression was used to conduct univariate prediction analysis on the 3 factors that were found to be significant in the single predictive variable analyses. Only 1 factor was independently related to nonseizure-free outcomes after surgical treatment: TIST (*P* = .035; Table 3).

**Table 3:** Multivariate analysis of risk factors of seizure recurrence.

Variables	*β*	*P* value	OR	95% CI
Focal to bilateral tonic-clonic seizures	1.732	.063	5.394	1.975−11.650
Simple motor signs	0.415	.648	1.478	0.291−8.162
Temporal-insular spreading time (ms)	−0.184	.035	0.797	0.625−0.978

CI = confidence interval, OR = odds ratio.

**Figure 2.**
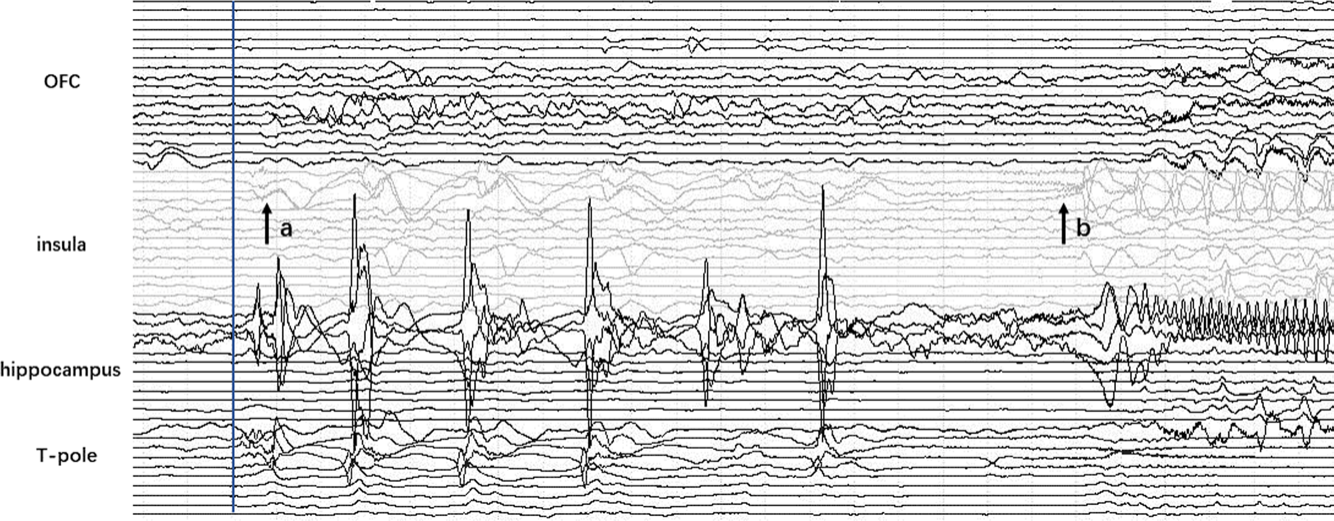
The receiver operating characteristic curve for the temporal-insular spreading time (TIST). Sensitivity is listed on the *y*-axis, and 1 − specificity is listed on the *x*-axis. The area under the curve was 0.788 suggesting that the TIST is a reliable predictor for the seizure-free outcome after surgery.

ROC curves were used to evaluate the continuous variables of TIST and to determine the cutoff values. The results of the ROC curve for TIST: a cutoff value of 400 milliseconds, and the area under the curve was 0.788 (Fig. 2).

The patient group was further divided into 2 subgroups according to TIST: TIST: 0 to 400 milliseconds and TIST > 400 milliseconds. The rates of seizure-free (Engle class I) were 33.3 % (3/9) and 62.5 % (20/32) in the 2 groups, respectively; the difference was statistically significant (*P* = .001; Table 4).

**Table 4:** Postoperative seizure outcomes and the comparation between groups with different TIST.

Postoperative seizure outcome (Engle class)	All patient, N = 41 (%)	Insular involved TLE patients, N = 39 (%)	TIST ≤ 400 ms, N = 9 (%)	TIST > 400 ms, N = 32 (%)	*P* value
Engle class I	23 (56.1)	21 (53.8)	3 (33.3)	20 (62.5)	.001
IA	19 (46.5)	17 (43.4)	0	16 (50.0)	–
IB	2 (4.8)	2 (5.2)	1 (11.1)	2 (6.2)	–
IC	1 (2.4)	1 (2.6)	0	1 (3.1)	–
ID	1 (2.4)	1 (2.6)	2 (22.2)	1 (3.1)	–
Engle class II–IV	18 (43.9)	18 (33.3)	6 (66.7)	12 (37.5)	–
II	11 (26.8)	11 (27.3)	3 (33.3)	8 (25.0)	–
III	4 (9.8)	4 (3.0)	2 (22.2)	2 (6.3)	–
IV	3 (7.3)	3 (3.0)	1 (11.1)	2 (6.3)	–

TIST = temporal-insular spreading time; TLE = temporal lobe epilepsy.

### 3.5. Surgical treatment and outcome

All the 41 patients underwent resective surgery; all the patients underwent ATL including the anterior hippocampus, amygdala, parahippocampal gyrus, and the temporal pole. Postoperative pathologies revealed HS in 30 patients, focal cortical dysplasia type I in 4 patients, and focal cortical dysplasia type IIIa in 7 patients.

Surgical outcomes were assessed according to the Engel classification.^[12]^ After at least 1-year follow-up (a mean period of 14.0 months after surgery), 23 (56.1%) of the 41 patients had an Engel I class, 11 (26.8%) cases reached Engel II, 4 (9.8%) reached Engel III, and 3 (7.3%) cases were Engel IV with less improvement or no effect of treatment (Table 4).

## 4. Discussion

Surgical treatment of refractory TL epilepsy has proven to be safe and effective in recent decades. However, a certain percentage of surgical failures in ATL remain insurmountable. The seizures occurring after the ATL may be due to the epileptic focus is not fully resected or a subordinate epileptic focus existed and gradually became the seizure onset area. The failure of seizure control could be explained by a more widespread epileptogenic network involving the TL, rather than a single localized focus. Improvements in SEEG recordings have provided a methodology to investigate the role of the insular cortex in the origin of TLE seizures. Nevertheless, it is difficult to evaluate the extent of insular involvement in TL seizures,^[11,12]^ and the factors influencing surgical outcomes remain uncertain. Our study demonstrated a predictor not previously proposed for surgical failure of ATL, and the main findings of this study are as follows:

1. Very early insular involvement may influence postoperative seizure outcome;
2. A TIST of 400 milliseconds tended to be an indicator for classifying very early or early insular involvement.

As demonstrated by previous studies, the phenomenon of ictal discharges invading the insular during TL seizures are common; the reported proportion of insular involvement ranges from 50% to 100% in the TL seizures.^[13,14]^ In our study, 97 of 103 seizures (39/41 patients) propagated to the insula. Eighteen of the 41 patients (43.9%) presented “TL” seizures resistant to a temporal lobectomy. Therefore, the results indicate that insular involvement alone cannot predict postoperative seizure outcomes. Another important phenomenon observed was that the TIST varied greatly among patients, and previous studies have not systematically evaluated the time delay of insular involvement in TL seizures. This may be significant in estimating the prognosis of seizure control after surgery. Isnard et al^[11]^ observed early insular propagation. Two patients with early insular propagation were not seizure-free after ATL. However, limited by the small number of cases, the prognostic significance of early insular involvement has not yet been evaluated. In our research, an ROC curve for TIST was calculated, and the cutoff value was 400 milliseconds, which provides potential guidance for the classification of insular involvement. We further grouped the patients according to the spreading time delay as follows: TIST ≤ 400 milliseconds and TIST > 400 milliseconds. The difference in the rates of postoperative seizure-free status was significant between the 2 groups. This implies that the TIST may be a candidate for predicting surgical outcomes.

Some characteristic symptoms accompanying TL seizures usually suggest insular involvement, such as somatosensory, motor, or visceral symptoms and sensation of asphyxia.^[15]^ In this case, scalp EEG is unreliable for identifying insular discharge. If focal simple motor signs appear early during temporal seizures, temporal-insular-opercular involvement should be considered.^[16]^ Simple motor signs usually manifest as limbs tonic or versive of the eyes and head, which may reflect a propagation of the discharge to the motor and premotor cortices.^[17,18]^ Our SEEG data revealed that simple motor signs usually reflect activation of fronto-insular cortical regions and not only the characteristic manifestation of insular epilepsy; similar observations were described in studies of Obaid et al and Martinez-Lizana et al.^[19,20]^ Under these circumstances, invasive SEEG is necessary to identify the epileptogenic and symptomatogenic zones. In our study, 5 patients had simple motor signs that appeared at the beginning of the onset discharges; their insular-opercular cortex invaded to varying degrees. However, the multivariate analysis showed that the simple motor signs did not have a significant association with the failures of temporal lobectomy. These signs can be significant in determining who should undergo intracerebral recording before surgery.

Using various methodologies, connections between the insula, temporal pole, and amygdala-hippocampal structure have been demonstrated in previous studies. The anterior insula mainly projects to the anterior cingulate areas, the entorhinal cortex, and the periamygdaloid cortex; in contrast, the mesial temporal structures (amygdala, hippocampus, and entorhinal cortex) have fewer projection fibers with the insula.^[21–23]^ Our SEEG recordings showed that ictal temporal discharges propagated to the insular cortex mainly followed 3 spreading routes and the TIST varied across different routes. The external temporal cortex, including the temporal pole, had a shorter spreading time to the insula, whereas the mesial structures (amygdala and hippocampus) had a much longer time span of insular spreading. These findings support the hypothesis of the presence of a direct pathway for rapid epileptic propagation between the temporal pole and the insular cortex. The fast epileptic discharges can propagate through the “temporoinsular projection system,” which forms a white matter pathway for direct ictal spread from the anterior TL structures toward the insular cortex.^[24]^ The TL seizures frequently propagate to the insula and have symptoms and signs of insular epilepsy; the rapid discharges spread from the TL to the insula is the presumed mechanism.

The duration of preoperative epilepsy on postoperative outcome is discussed controversially in previous studies.^[25–27]^ In general, a longer history of seizures may lead to the formation of secondary epileptogenic networks and thus result in the failure of epilepsy surgery. However, we did not find such associations in our study. The possible reasons for this result may be associated with several factors, such as pathological types, location of temporal lesions, and whether the HS as an only temporal lesion. In subsequent studies, we will continue to focus on prognostic factors of postoperative seizure outcomes in patients with different pathological types.

Inevitably, there were limitations to our study. For example, it remains a retrospective study, and the classification of insular involvement was based on a postoperative review of SEEG results, resulting in the possibility of bias in data analysis. In addition, the number of patients in the subgroup with very early insular involvement was relatively small is another limitation, and further studies are needed to obtain more reliable conclusions.

## 5. Conclusions

Our results suggest that the TIST can be an independent predictor of surgical outcome in TLE patients with insular involvement. The very early insular involvement in TLE may be associated with poorer seizure outcomes after ATL. Our findings may be helpful for estimating whether the ATL is adequate for patients who were identified as “very early insular involvement” TLE. These results might also be valuable for the prognosis of patients with temporal-insular SEEG recordings and temporal lobectomy.

## Author contributions

Conceptualization: Xi Zhang.

Data curation: Cuiping Xu, Jin Zhu, Kai Ma, Xiaoming Yan.

Investigation: Guojun Zhang, Tao Yu, Xi Zhang.

Methodology: Xi Zhang.

Resources: Runshi Gao.
